# Contemporary Evidence for Optimization of Robotic Radical Prostatectomy Outcomes Using Advanced Imaging Techniques

**DOI:** 10.3390/jcm15041631

**Published:** 2026-02-21

**Authors:** Gary K. Shahinyan, David S. Finley

**Affiliations:** Department of Urology, Kaiser Permanente Los Angeles Medical Center, Los Angeles, CA 91605, USA

**Keywords:** robotic-assisted radical prostatectomy, prostate cancer, bi-parametric MRI, multiparametric MRI, PSMA PET/CT, artificial intelligence, intraoperative imaging, surgical margins, nerve-sparing surgery

## Abstract

**Background/Objectives:** Robotic-assisted radical prostatectomy (RARP) is a standard treatment for localized and locally advanced prostate cancer; however, optimizing oncologic control while preserving urinary continence and erectile function remains challenging. Advances in preoperative imaging, molecular diagnostics, artificial intelligence (AI), and intraoperative assessment have the potential to refine surgical planning and execution. This review summarizes contemporary evidence on advanced imaging and intraoperative technologies used to optimize RARP outcomes. **Methods:** A narrative literature review was conducted of English-language studies published between 2015 and 2025 using PubMed/MEDLINE, Scopus, and Google Scholar. Studies evaluating multi-parametric and bi-parametric MRI, prostate-specific membrane antigen-based positron emission tomography/computed tomography (PSMA PET/CT), AI-assisted tumor modeling, and intraoperative histologic or molecular imaging techniques in the context of robotic-assisted radical prostatectomy were included. Evidence from randomized controlled trials, prospective and retrospective studies, technical feasibility reports, and expert consensus statements was reviewed. **Results:** MRI remains central to anatomic mapping and local staging but consistently underestimates true tumor extent, with implications for margin control. AI-assisted platforms improve tumor contouring accuracy and may meaningfully influence surgical decision-making. PSMA-based imaging enhances detection of extra-prostatic extension and nodal disease and shows early promise for ex vivo and intraoperative guidance. Intraoperative margin assessment techniques are supported by randomized evidence demonstrating improved functional outcomes without compromising short-term oncologic safety and emerging digital histologic technologies offer scalable alternatives for real-time margin evaluation. **Conclusions:** Integration of advanced anatomic, molecular, and intraoperative imaging technologies represents an evolving multimodal paradigm in RARP. Combined use of MRI, PSMA-based imaging, AI-assisted modeling, and rapid histologic assessment may enable more precise, individualized surgery that balances oncologic control with functional preservation. Further validation is required to define optimal implementation in routine clinical practice.

## 1. Introduction

Robotic-assisted radical prostatectomy (RARP) has become the predominant surgical approach for the management of localized and locally advanced prostate cancer, offering improved visualization, dexterity, and reproducibility compared with open techniques. Despite these advances, surgeons continue to face the challenge of balancing oncologic control with preservation of urinary continence and erectile function. Positive surgical margins, early functional recovery, and long-term quality-of-life outcomes remain closely linked to the accuracy of preoperative risk stratification and the precision of intraoperative decision-making, particularly during apical dissection and nerve-sparing.

Advances in preoperative and intraoperative imaging have reshaped contemporary prostate cancer surgery. Multiparametric magnetic resonance imaging (mpMRI) remains central to local staging and anatomic delineation, while prostate-specific membrane antigen-based positron emission tomography/computed tomography (PSMA PET/CT) has expanded the ability to detect clinically significant disease beyond the limits of conventional imaging. However, growing evidence demonstrates that these modalities may underestimate true tumor extent and fail to identify microscopic or infiltrative disease, with important implications for margin control. In response, artificial intelligence-assisted (AI) imaging platforms and intraoperative assessment techniques have emerged to enhance tumor modeling, refine surgical planning, and provide real-time feedback during nerve-sparing and margin-directed resections, signaling a shift toward precision-guided surgery.

This review examines contemporary evidence supporting the integration of advanced imaging, AI-assisted modeling, and intraoperative assessment techniques in RARP, with a focus on optimizing oncologic precision while preserving functional outcomes.

## 2. Materials and Methods

A narrative literature search was conducted to identify relevant studies published between 2015 and 2025 addressing imaging- and technology-assisted optimization of RARP. Searches were performed in major electronic databases, including PubMed/MEDLINE, Scopus, and Google Scholar, using combinations of controlled vocabulary and free-text keywords. Representative search terms included “multiparametric MRI” AND “radical prostatectomy”, “biparametric MRI” AND “radical prostatectomy”, “PSMA PET/CT” AND “radical prostatectomy”, “artificial intelligence” AND “prostate cancer surgery”, and “intraoperative histology” AND “robotic prostatectomy”, along with other relevant keyword combinations related to advanced imaging and intraoperative technologies.

Searches were limited to English-language publications and filtered by publication date. Reference lists of selected articles were manually reviewed to identify additional relevant studies not captured by the database search. Evidence from randomized controlled trials, prospective studies, retrospective cohorts, technical feasibility studies, and expert consensus statements was considered. Given the narrative design of this review, no formal systematic review methodology or meta-analysis was performed.

## 3. Results

### 3.1. Utility of MRI, PSMA PET-CT, and Frozen Section Histology in Intraoperative Planning

#### 3.1.1. MRI

mpMRI is integral to the preoperative assessment and intraoperative planning of RARP. Prostate anatomy is highly variable, particularly at the bladder neck and apex [1,2]. A detailed understanding of prostate size, shape, and three-dimensional tumor location is essential not only for surgical preparation but also for informed shared decision-making with patients regarding realistic expectations for functional outcomes. The presence of a median lobe, for example, signals potential challenges to bladder neck sparing, which has a direct and reproducible impact on early continence outcomes [3]. Beyond lesion localization and staging, mpMRI provides critical information regarding tumor size, extracapsular extension, and involvement of potential surgical margins—features that strongly influence intraoperative decision-making and help mitigate the risks of positive surgical margins (PSMs), urinary incontinence, and erectile dysfunction [4].

Recent evidence supports the predictive utility of MRI-based risk stratification for reducing PSMs. Xu et al. (2023) proposed a grading system that integrates tumor capsular contact length, extracapsular extension, and lesion location to estimate PSM risk preoperatively, demonstrating good discriminatory accuracy and potential clinical utility for guiding nerve-sparing decisions and resection planes [5]. Similarly, Quentin et al. (2022) showed that preoperative mpMRI parameters—including length of capsular contact (LCC) and distance to the membranous urethra—can identify tumors at higher risk for PSMs, underscoring the importance of close radiologic–surgical correlation in tailoring operative strategies [6].

Despite these advances, mpMRI remains constrainedin its ability to precisely quantify true tumor volume, particularly for small or low-grade lesions. Priester et al. conducted one of the most methodologically rigorous evaluations of mpMRI accuracy by using patient-specific 3D-printed molds to achieve precise co-registration between preoperative mpMRI and whole-mount prostate pathology in 114 men. Suspicious regions of interest (ROIs) were contoured on T2-weighted images, and custom molds enabled exact alignment of sliced prostate specimens with corresponding MRI slices, allowing true three-dimensional comparison of tumor geometry. Among 222 tumors identified on whole-mount pathology, 118 had corresponding MRI ROIs. For these tumors, the mean ROI volume was 0.8 cc with a mean longest 3D diameter of 17 mm, whereas matched pathologic tumors had a mean volume of 2.5 cc and the longest diameter of 28 mm. The median tumor extended 13.5 mm beyond the MRI-defined contour, and approximately 80% of tumor volume lay outside the ROI boundaries. Underestimation was most pronounced along the base–apex axis, while size estimation was most accurate in the axial plane.

Consistent with these findings, Sorce et al. (2022) similarly reported systematic underestimation of lesion volume on mpMRI compared with histopathologic measurements [7]. Together, these data provide compelling quantitative evidence that mpMRI incompletely characterizes the geographic extent of the lesion, with important implications for defining safe resection planes—particularly in regions such as the apex and along the neurovascular bundles, where millimeter-level precision is critical for margin control.

**Figure 1:**
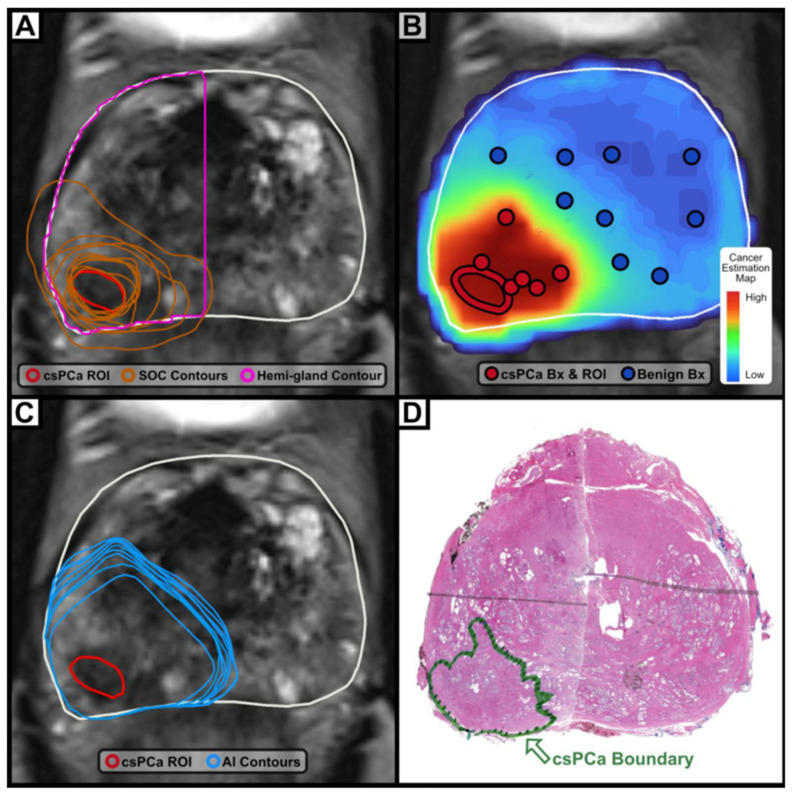
This figure from Mota et al. demonstrates the comparison between standard of care (cognitive) vs. AI-assisted mapping for prostate contouring. ROI marking (red) in addition to SOC (orange) and hemi-gland (pink) contours for focal therapy (**A**). AI-generated cancer estimation map (**B**), AI-generated contours for focal therapy (**C**), and wholemount pathology boundary (**D**). Bx = biopsy, SOC = standard of care, cxPCa = clinically significant prostate cancer, ROI = region of interest [9].

MRI’s role in volumetric analysis has been enhanced by emerging computational approaches. Yilmaz et al. (2024) demonstrated that deep learning algorithms applied to bpMRI improve volumetric accuracy and correlate more closely with histopathologic tumor mapping than radiologist assessment alone [8]. Within the AI domain, decision-support tools such as Unfold AI (Avenda Health, Culver City, CA, USA) have further advanced focal therapy and RARP planning by integrating tracked fusion biopsy data with mpMRI and pathology to more precisely capture the true tumor volume. In a multi-reader, multi-case study at UCLA, investigators evaluated whether Unfold AI improved prostate cancer contouring compared with standard-of-care (SOC) cognitive delineation as demonstrated in Figure 1 [9]. Fifty radical prostatectomy cases suitable for focal therapy were contoured by ten physicians (seven urologists and three radiologists), first using SOC methods and then with AI assistance after a four-week washout period. Whole-mount histopathology served as the reference standard. AI-assisted contours demonstrated significantly higher balanced accuracy—defined as the mean of voxel-based sensitivity and specificity—compared with SOC and hemi-gland contouring (84.7% vs. 67.2% and 75.9%, respectively; *p* < 0.001). The negative margin rate improved from 1.6% with SOC to 72.8% with AI assistance (*p* < 0.001), while average contouring time decreased from 3.5 to 2.0 min (*p* < 0.001). Notably, treatment recommendations changed in 42% of cases following AI-assisted review.

**Figure 2:**
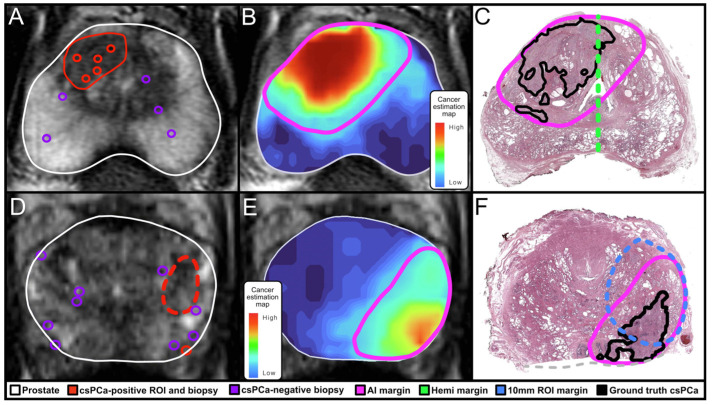
The figure above from Priester et al. demonstrates the function and use of the AI technology used by Unfold AI. The first column (**A**,**D**) shows the MRI, biopsy and projected ROI based on MRI. Images (**B**,**E**) demonstrate the AI-generated margins and heatmap and columns (**C**,**F**) show the correlated whole-mount pathology along with segmentation of clinically significant prostate cancer [10].

In a complementary analysis, Priester et al. validated an AI-driven cancer estimation model using an independent whole-mount prostatectomy dataset. AI-generated margins demonstrated substantially higher sensitivity (97%) than conventional MRI-defined regions of interest (ROIs) (37%) and outperformed hemigland-based margins while maintaining comparable treatment volumes [10]. Default AI margins achieved negative margins in 80% of all clinically significant cancers and 90% of index lesions, markedly exceeding hemigland performance (56% and 66%, respectively), thereby highlighting the limitations of uniform or hemisphere-based treatment strategies. A representative correlation of the AI heat map and biopsy results can be seen in Figure 2. Collectively, these findings suggest that AI-derived, multimodal models can meaningfully enhance focal therapy planning by more accurately capturing true tumor extent, particularly in cases involving midline extension or MRI-invisible satellite foci. Beyond focal therapy, the integration of AI-based tumor modeling may also play an increasingly important role in optimizing surgical planning for RARP.

Future incorporation of AI tools may further refine risk estimation and margin delineation, enabling increasingly personalized surgical strategies that balance oncologic control with nerve sparing for preservation of erectile function and continence. Conversely, a subset of men with negative or low-risk preoperative mpMRI findings may still harbor clinically significant disease. Yaxley et al. (2021) highlighted this limitation through histologic evaluation of 1197 robot-assisted prostatectomy specimens, demonstrating nontrivial rates of tumor presence even within MRI-negative lobes and cautioning against overreliance on MRI alone for operative planning [11]. These findings reinforce MRI’s role as an adjunctive—rather than definitive—tool for surgical mapping, a role that may be further strengthened through integration with AI and machine learning technologies. Importantly, no randomized controlled trials or prospective outcome-linked studies have yet demonstrated that AI-assisted tumor modeling improves hard surgical endpoints such as positive surgical margin rates, urinary continence, or erectile function following RARP. As such, proposed clinical benefits—including improved margin control, optimized nerve sparing, and reduced functional morbidity—remain inferential rather than proven. These tools should therefore be regarded as advanced decision-support systems that augment, but do not replace, surgeon judgment.

Recent evidence also suggests that evolving MRI protocols may influence the cost, accessibility, and utility of imaging for surgical planning. The PRIME trial, a prospective, multicenter noninferiority study of 490 biopsy-naïve men, demonstrated that bpMRI, consisting of T2-weighted and diffusion-weighted imaging without dynamic contrast enhancement, was noninferior to standard mpMRI for the detection of clinically significant prostate cancer [12]. Assessment of extracapsular extension and involvement of key anatomic structures—including the bladder neck, urethral sphincter, and seminal vesicles—was highly concordant between imaging approaches, and the addition of contrast altered treatment eligibility or surgical planning in only 3–4% of cases. Although PRIME was not designed to evaluate oncologic or functional outcomes, these results suggest a potential shift toward bpMRI in prostate cancer screening and preoperative planning, with meaningful savings in time, cost, and gadolinium exposure.

Given that most studies cited above were performed using mpMRI, further investigation is needed to clarify the role of bpMRI in preoperative planning prior to RARP. While the PRIME trial supports the noninferiority of bpMRI for detecting clinically significant prostate cancer, its findings do not imply that bpMRI should universally replace mpMRI for all patients undergoing RARP. In clinical practice, bpMRI appears sufficient for prostate cancer workup and routine preoperative planning in many men, particularly when the primary goals are lesion localization and risk stratification. However, mpMRI may remain as the preferred modality in selected scenarios—such as suspected extracapsular extension, apical or bladder neck involvement, prior discordant imaging or biopsy findings, or complex surgical decision-making—where contrast-enhanced sequences may provide incremental anatomic or staging information.

Collectively, the current literature underscores MRI as a powerful tool for individualized surgical planning. Its primary strengths lie in local staging and risk prediction, while limitations persist in the detection of small, apical, or infiltrative lesions. Continued advances in image quality, magnet strength and AI-driven modeling are expected to enhance MRI’s intraoperative relevance

#### 3.1.2. PSMA PET/CT and PSMA Based Imaging 

Prostate-specific membrane antigen (PSMA)-targeted imaging has emerged as an important adjunct in both preoperative and intraoperative planning for radical prostatectomy. Its high tumor-to-background discrimination and ability to delineate clinically significant disease beyond the boundaries of conventional MRI have important implications for margin control, nerve preservation, and—most notably—nodal assessment.

High-level evidence supporting PSMA PET/CT in high-risk prostate cancer emerged from the proPSMA randomized controlled trial, a prospective multicenter study demonstrating significantly greater staging accuracy for PSMA PET/CT compared with conventional CT and bone scintigraphy, along with fewer equivocal findings and more frequent management changes [13]. Although this trial was not designed to assess surgical outcomes, it established PSMA PET/CT as a superior staging modality and has informed its incorporation into major guidelines as a complementary tool for preoperative risk stratification and surgical planning in high-risk disease. This provides important context for ongoing efforts to integrate PSMA-based technologies into intraoperative workflows, which remain supported primarily by early-phase and observational studies.

Intraoperative application of PSMA-based PET/CT shows promise for real-time surgical guidance. In a prospective pilot study of seven high-risk patients (18 lesions), Moraitis et al. demonstrated that ex vivo [^18^F]PSMA-1007 PET/CT, combined with iterative threshold-based segmentation, detected macroscopic PSMA-positive surgical margin involvement with 83% sensitivity, 100% specificity, and 94% overall accuracy. PET-derived margin measurements correlated closely with whole-mount histopathology (r = 0.90) [14]. These findings support the feasibility of PSMA-guided radiologic assessment immediately following gland removal. Although detection is currently limited to macroscopic PSMA-avid foci and cannot replace histologic evaluation of microscopic disease, these early results suggest that intraoperative PSMA imaging—whether applied ex vivo or eventually integrated into real-time surgical environments—may evolve into a valuable adjunct for confirming critical resection planes and informing nerve-sparing decision-making.

**Figure 3:**
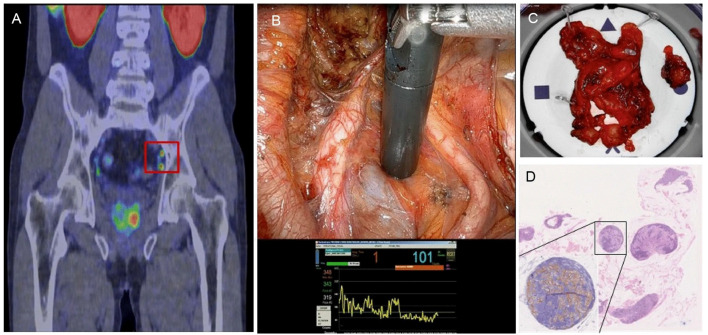
PSMA-PET shows pathologic uptake in the whole prostate in addition to suspicious signs of extra-capsular extension and two PSMA-positive left obturator lymph nodes (red square) (**A**). This patient underwent radical prostatectomy with extended pelvic lymph node dissection (PLND. After IV injection of 90 MBq of 68 Ga-PSMA-11 administered in the surgery theatre, a DROP-IN beta probe was used during surgery with both the physical probe and the on-screen output measurement (**B**). The gross specimen and histopathological analysis are shown in (**C**) and (**D**), respectively [15].

Beyond specimen-based PET/CT, broader developments in PSMA radio-guided surgery (PSMA-RGS), which does not require an extracted specimen, further underscore the expanding role of molecular imaging during surgery. In a recent expert review, Muraglia et al. summarized clinical experience with both gamma-emitting and beta-emitting probes for PSMA-targeted radio-guidance and highlighted strong concordance between PSMA-RGS-detected lymph nodes and histopathologic findings in early-phase trials [15]. PSMA-RGS demonstrated high sensitivity and specificity for identifying metastatic lymph nodes, particularly for lesions larger than 3–4 mm, while size-dependent limitations persisted for microscopic disease. Importantly, emerging technologies—including drop-in beta probes (Figure 3 below), luminescence imaging, and PET/CT specimen imagers—have demonstrated feasibility within the robotic milieu, offering enhanced tumor-to-background contrast and improved interrogation of superficial tumor deposits [16]. Although these approaches currently complement rather than replace standard pelvic lymphadenectomy or frozen-section analysis, they reflect an evolving ecosystem of PSMA-based intraoperative tools that may ultimately integrate into real-time surgical workflows to augment anatomic dissection and refine oncologic precision.

**Figure 4:**
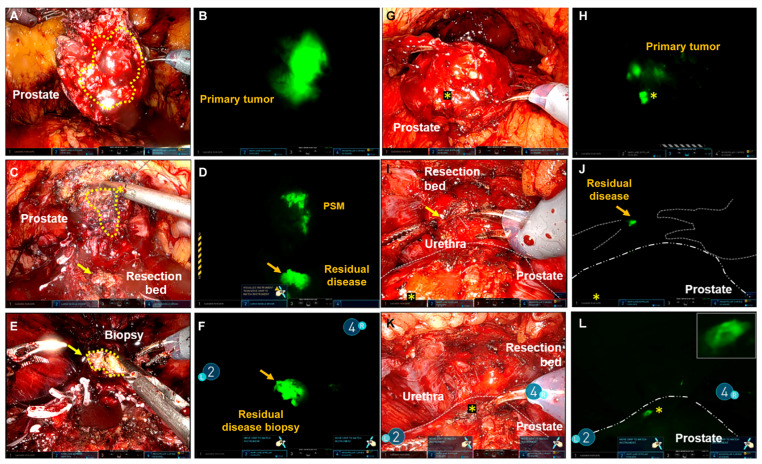
Intraoperative exploratory efficacy of IS-002 during robotic prostatectomy demonstrating detection of primary tumor, positive surgical margins, and residual disease. Example 1 (**A**–**F**): White light and Firefly imaging of the excised prostate (~23 h after IS-002 injection) showing fluorescence within the primary tumor, as well as intraoperative identification of fluorescent tissue corresponding to pathology-confirmed positive surgical margins and bladder neck invasion. Example 2 (**G**–**L**): Firefly imaging (~24 h after IS-002 injection, 100 µg/kg) demonstrating focal fluorescence within the primary tumor and detection of residual disease in the anterior apical resection bed near the urethra that was not apparent under white light and was confirmed as adenocarcinoma on biopsy. Gold stars represent the transected prostate tumor margin during surgery.

**Figure 5:**
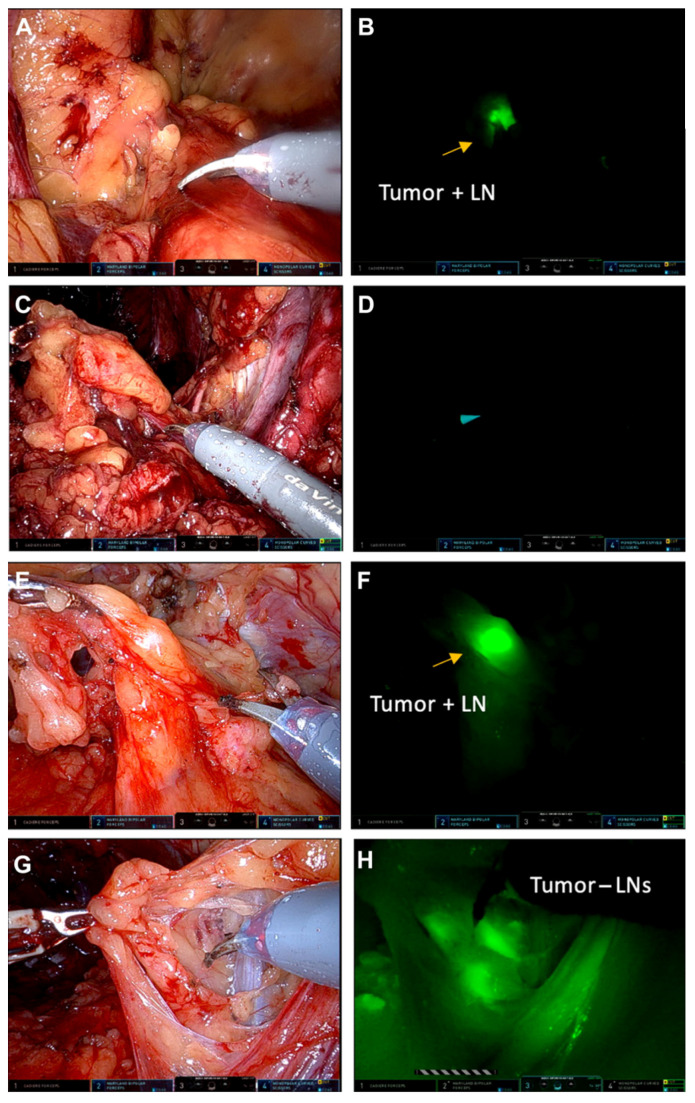
IS-002 fluorescence in tumor-positive and tumor-negative lymph nodes during robotic prostatectomy. White light and corresponding Firefly images demonstrate PSMA-targeted fluorescence in tumor-positive lymph nodes compared with minimal background signal in contralateral tumor-negative nodes. (**A**,**B**) tumor-positive lymph node following IS-002 injection (25 µg/kg) and (**C**,**D**) contralateral tumor-negative lymph node package. Different patient: (**E**,**F**) tumor-positive lymph node following IS-002 injection (150 µg/kg) and (**G**,**H**) contralateral tumor-negative lymph node package. LN, lymph node.

A recent first-in-human evaluation of intraoperative live imaging using a near-infrared, PSMA-targeted fluorescent small molecule (IS-002) demonstrated the feasibility of real-time optical molecular imaging during robotic prostatectomy [17]. In this study of 24 men with high-risk prostate cancer, IS-002 enabled intraoperative visualization of primary tumors, positive surgical margins, and previously unrecognized residual disease within the resection bed. Notably, fluorescence imaging identified locoregional or residual disease in 7 of 24 patients (29%), including cancers not appreciated under standard white-light visualization. The use of intraoperative IS-002 is depicted well in Figure 4 and Figure 5. In one case, a lesion detected by fluorescence was confirmed on frozen section analysis despite the absence of a corresponding positive margin on final whole-mount histopathology—a finding the authors suggest may reflect limitations of routine 5 mm pathologic sectioning. At the optimal dose (25 µg/kg), this technique achieved a negative predictive value of 100% for detecting disease in the resection bed, with clear tumor-to-background demarcation.

**Figure 6:**
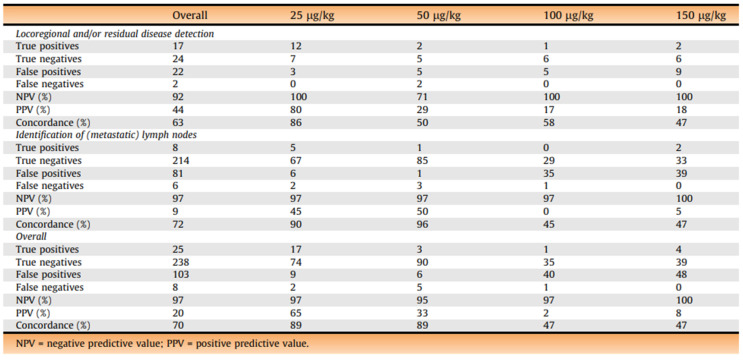
The figure above demonstrates the diagnostic accuracy with the four different dosing protocols ranging from 25–150 µg/kg. Higher doses demonstrated higher false positive rates, consistent with the concern of elevated urinary clearance and high, non-specific signal accumulation.

IS-002 enabled fluorescence-based identification of PSMA-positive lymph nodes and intraoperative detection of nodal disease in five patients, achieving a negative predictive value of 97% at the lowest administered dose. However, clear dose-dependent trade-offs were observed. Higher doses (100–150 µg/kg) produced greater background fluorescence—attributed to ongoing urinary clearance and nonspecific tissue uptake—leading to reduced specificity and markedly higher false-positive rates. In contrast, the 25 µg/kg dose provided the most favorable balance between tumor signal/background and was therefore identified as the optimal intraoperative dosing window as can be seen in Figure 6. Notably, fluorescence guidance revealed sites of disease not detected by preoperative PSMA PET/CT, including urethral-adjacent extension and levator ani invasion, highlighting its potential to complement both mpMRI and nuclear PSMA imaging.

Pharmacokinetically, IS-002 demonstrated predictable behavior after intravenous administration across the tested dose range (25–150 µg/kg). Plasma levels peaked within minutes and exhibited an elimination half-life of approximately 5–8 h and primary clearance through the kidneys, supported by formal correlations with renal function and by dose-dependent urinary discoloration [17]. No hepatic clearance pathway or association with body mass index was reported. These properties directly informed the surgical workflow: radical prostatectomy with extended pelvic lymph node dissection was performed approximately 24 h after administration, corresponding to several drug half-lives. This interval allows substantial systemic washout while preserving tumor-associated fluorescence, thereby minimizing background signal from circulating tracer and urinary excretion. Together, these findings support a next-day, low-dose fluorescence-guided strategy that maximizes intraoperative specificity while maintaining clinically useful tumor visualization.

Building on this paradigm, prior fluorescence-guided oncologic interventions provide valuable context for the evolution of intraoperative imaging. In colorectal liver metastasectomy, indocyanine green (ICG) has been used to generate a peritumoral fluorescent rim, enabling real-time assessment of resection margins under near-infrared illumination and improving intraoperative confidence in achieving complete tumor clearance [18]. Similar principles have been applied in robotic partial nephrectomy, where ultra-low-dose ICG exploits differential perfusion to distinguish renal tumors from adjacent normal parenchyma, thereby facilitating precise tumor localization and parenchymal preservation [19,20]. Despite these successes, translation of ICG-based near-infrared fluorescence to RARP has been limited by the absence of a reliable, tumor-specific signal on the prostate surface, restricting its utility for margin assessment or mapping of extracapsular disease. These limitations have driven efforts to move beyond nonspecific perfusion dyes toward molecularly targeted fluorescent agents—a direction long anticipated with the development of next-generation imaging platforms capable of integrating such probes into routine robotic surgery.

Taken together with emerging hybrid tracers and PSMA-radioguided techniques, IS-002 represents a maturing class of visual and molecular navigation tools with the potential to significantly enhance intraoperative decision-making, particularly in high-risk radical prostatectomy.

#### 3.1.3. Intra-Operative Histologic Review

Intraoperative histologic assessment remains one of the most reliable methods for balancing oncologic control with functional preservation during radical prostatectomy. However, its utility is constrained by the finite amount of tissue that can be excised, extracted through a port, processed, and interpreted rapidly while the patient remains anesthetized. The NeuroSAFE (Neurovascular Structure-Adjacent Frozen Section Examination) technique, first described by Schlomm and colleagues in 2012, addresses this limitation by enabling frozen-section analysis of the entire posterolateral surface of the prostate to assess margin status adjacent to the neurovascular bundles [21]. According to the authors, this approach permits immediate intraoperative adjustment of the degree of nerve sparing. NeuroSAFE was initially applied for oncologic navigation in 5392 open radical prostatectomies. By systematically cleaving and evaluating the entire neurovascular bundle-adjacent prostate surface at 5 mm intervals, NeuroSAFE allows preservation of nerve tissue when margins are histologically negative and facilitates secondary resection of the bundle when ontologically necessary.

The first randomized controlled evaluation of this approach—the multicenter NeuroSAFE PROOF phase III trial—now provides high-level evidence supporting its functional benefit [22]. In this patient-blinded trial of 381 men with good baseline erectile function, NeuroSAFE-guided RARP, using an Alexis port for specimen extraction, significantly improved erectile function at 12 months compared with standard RARP (mean IIEF-5 score 12.7 vs. 9.7; adjusted mean difference +3.18, 95% CI 1.62–4.75; *p* < 0.0001). Positive surgical margin rates were 35% in the NeuroSAFE group versus 29% in the standard group, and 44% of secondary resections contained cancer. NeuroSAFE also increased bilateral nerve-sparing rates (82% vs. 56%) and reduced early urinary symptoms, with significantly lower 3-month ICIQ scores (adjusted mean difference −1.41; *p* = 0.006). These functional improvements were not associated with a meaningful increase in adverse oncologic outcomes: PSA persistence or biochemical recurrence occurred in 9% of NeuroSAFE patients compared with 6% in the standard group at 12 months, a difference unlikely to reflect prognostic divergence given the short follow-up. Importantly, the greatest benefit was observed in men not initially considered candidates for bilateral nerve sparing, in whom NeuroSAFE improved IIEF-5 scores by nearly four points compared with standard RARP (interaction *p* = 0.028). Although NeuroSAFE added an average of 42 min to operative time and required specialized personnel, these results establish NeuroSAFE as the first technique proven in randomized testing to improve patient-reported erectile function and early continence outcomes after RARP.

Complementing these randomized data, Köseoğlu et al. (2023) evaluated the role of NeuroSAFE in the contemporary imaging context of mpMRI and PSMA PET/CT [23]. In a retrospective cohort of 208 men, NeuroSAFE significantly increased bilateral nerve-sparing rates (81% vs. 55%) and substantially reduced positive margin rates within nerve-preserved regions of interest (3.3% vs. 23.4%) compared with standard RARP. The benefit was most pronounced among patients with D’Amico high-risk disease and radiologic suspicion of extracapsular extension (iT3 on mpMRI or PSMA PET/CT), in whom NeuroSAFE achieved bilateral nerve sparing in 63% of cases and limited ROI-specific positive margins to 9%, compared with 30% and 80%, respectively, with standard RARP. Oncologic outcomes were also favorable: PSA persistence occurred in only 1.1% of NeuroSAFE patients versus 7.6% in the standard group, while biochemical recurrence rates were low and comparable (2.2% vs. 2.5%) over a median follow-up of 20–23 months. These findings highlight the additive value of intraoperative frozen-section assessment, even in an era of refined preoperative risk stratification using mpMRI and PSMA PET/CT.

**Figure 7:**
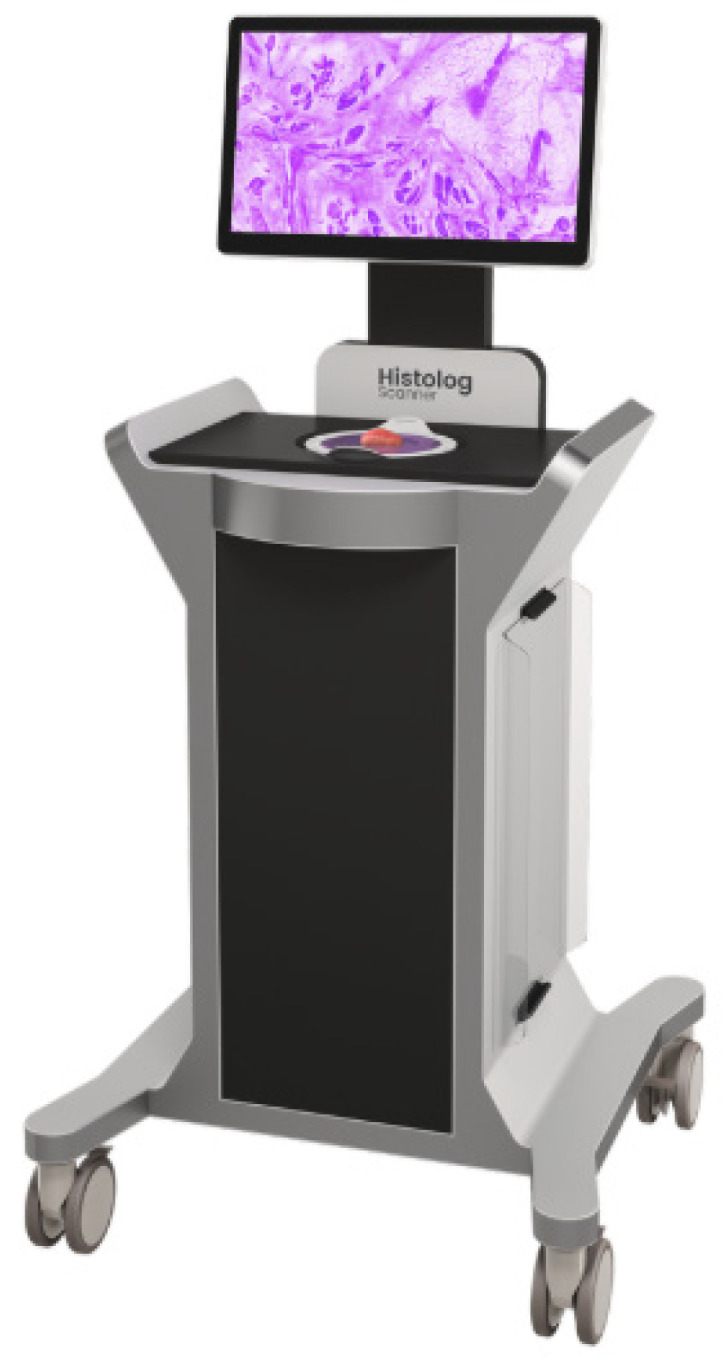
Histolog Scanner (SamanTree Medical, Liège, Belgium).

In parallel with advances in frozen-section workflows, digital ex-vivo fluorescence confocal microscopy (FCM) has emerged as a faster and less resource-intensive alternative for intraoperative margin assessment. The Histolog Scanner [Figure 7, (SamanTree Medical, Liège, Belgium)] enables high-resolution imaging of fresh prostate specimens without tissue sectioning, with image acquisition in approximately one minute per surface [24,25]. After limited training, surgeons can interpret FCM images intraoperatively as a decision-support tool. Early feasibility studies introduced the LaserSAFE technique, which involves en bloc scanning of the posterolateral capsule while preserving tissue integrity for subsequent conventional histopathologic analysis. The technique includes briefly immersing the prostate specimen in a fluorescent stain to enhance optical resolution and image detail. In a blinded evaluation of 31 RARP specimens, FCM interpretation achieved sensitivities of 73–91% and specificities of 94–100% for detecting positive surgical margins, with substantial inter-rater agreement compared with standard histologic sections [24,25].

A systematic review of fluorescence confocal microscopy (FCM) across solid tumors further supports its potential clinical role, reporting consistently high diagnostic accuracy in prostate applications, with sensitivities and specificities exceeding 80%. The review also highlighted important operational advantages, including rapid image acquisition, minimal tissue handling, and the capability for remote pathology review [26]. The ongoing multicenter IP8-FLUORESCE trial is expected to provide definitive, blinded, and adequately powered evidence regarding the diagnostic accuracy of the Histolog Scanner (seen in Figure 7) in radical prostatectomy, as well as its ability to detect clinically significant margins across all prostatic surfaces [27].

Although these digital techniques require further validation before widespread clinical adoption, they have the potential to address key limitations of NeuroSAFE—most notably its reliance on frozen-section infrastructure and specialized pathology staffing—and may expand access to real-time intraoperative margin assessment across diverse practice settings. As such, FCM-based systems such as the Histolog Scanner represent an important complement to traditional frozen-section workflows, with the capacity to broaden the availability of precision-guided, nerve-sparing prostatectomy.

Available evidence suggests that the surgeon learning curve for confocal microscopy is relatively short when supported by structured training. In a detailed implementation study in breast cancer surgery, surgeons achieved high diagnostic accuracy after approximately six hours of targeted image-based training, with rapid improvement following expert feedback and subsequent stable performance during routine clinical use [28]. No published studies have formally evaluated the learning curve for confocal microscopy in prostate cancer surgery, where tissue architecture, margin geometry, and clinical decision thresholds differ substantially from breast-conserving surgery.

However, a dedicated study developing an atlas of fluorescence confocal microscopy (FCM) images of prostatic and periprostatic tissues demonstrated that pathologists achieved reliable image interpretation after limited exposure, with consistent re-evaluation accuracy after a 90-day interval, suggesting a short and reproducible learning curve for prostate tissue assessment. From an implementation perspective, FCM may also offer substantial workflow advantages, with shortened procedure times compared with 50–63 min for NeuroSAFE frozen-section analysis, which may further facilitate adoption once surgeon-specific validation is established [29]. The generalizability of existing learning-curve evidence to surgeon-led confocal microscopy in prostate cancer remains uncertain and represents an important area for future prospective investigation.

## 4. Conclusions

The integration of advanced preoperative imaging, molecular diagnostics, and intraoperative histologic assessment is fundamentally reshaping surgical strategy in radical prostatectomy. At the present time, mpMRI remains the cornerstone modality for anatomic mapping and local staging, providing essential insight into prostate morphology, tumor location, extracapsular extension, and margin risk. However, its persistent limitations—particularly in accurately defining true tumor boundaries, detecting subtle apical disease, and identifying MRI-invisible foci—underscore the need for complementary technologies to refine surgical planning and execution.

Advances in AIhave begun to address some of these limitations by improving volumetric accuracy and tumor delineation, enabling more individualized preoperative planning. Nevertheless, interpretation of these data must be contextualized by important limitations in the underlying evidence base. Most mpMRI correlation studies evaluating tumor extent and margin risk are retrospective and derived from high-volume referral centers, introducing potential selection and spectrum bias. Similarly, AI-assisted tumor modeling studies are predominantly single-center and rely on retrospective datasets with same-institution or multi-reader validation designs, limiting assessment of generalizability across different scanners, MRI field strengths (1.5 T vs. 3.0 T), and acquisition protocols. Moreover, many AI studies emphasize technical performance metrics rather than downstream clinical or oncologic outcomes, and proposed benefits related to margin reduction or functional preservation therefore remain hypothesis-generating pending prospective, multicenter validation.

PSMA-based imaging further extends this paradigm by enabling molecular characterization of disease beyond the capabilities of conventional MRI. PSMA PET/CT has demonstrated clear advantages in staging accuracy for high-risk prostate cancer and is incorporated into clinical guidelines as an alternative to conventional imaging for initial staging. Its role in operative planning, however, remains indirect and constrained by spatial resolution limits and logistical considerations, and randomized evidence demonstrating improved surgical, oncologic, or functional outcomes is currently lacking. Emerging PSMA-targeted fluorescence agents offer real-time intraoperative visualization of tumor deposits, margins, and nodal disease, presenting a compelling framework for surgical navigation in selected high-risk cases. At present, these approaches remain investigational and are associated with additional regulatory, workflow, and cost considerations, with formal cost-effectiveness analyses still lacking. In addition, comparative cost-effectiveness data for PSMA-based imaging and intraoperative molecular guidance remain limited, particularly for resource-intensive and investigational approaches such as PSMA-targeted fluorescence imaging and fluorescence confocal microscopy, for which formal economic analyses have not yet been reported. The cost of established modalities, including MRI and PSMA PET/CT, also varies substantially across healthcare systems and institutions due to differences in reimbursement and resource availability, further complicating direct economic comparisons.

Despite rapid advances in imaging and molecular guidance, intraoperative histologic evaluation remains the most definitive method for margin assessment during radical prostatectomy. Techniques such as NeuroSAFE provide immediate, high-resolution evaluation of neurovascular-adjacent margins and are now supported by randomized controlled trial evidence demonstrating improved functional outcomes without compromising early oncologic safety. In parallel, digital confocal microscopy platforms such as the Histolog Scanner represent a promising, tissue-preserving alternative that may broaden access to real-time margin assessment in settings where frozen-section infrastructure is limited, although surgeon-specific validation in prostate cancer and more robust data and protocols for intra-operative implementation are still evolving.

Current international guidelines, including those from the EAU and NCCN, reflect this evidence hierarchy. mpMRI remains the preferred MRI modality for prostate cancer evaluation, while biparametric MRI is increasingly recognized as an acceptable alternative in selected scenarios based on recent noninferiority data. PSMA PET/CT is recommended as an appropriate staging modality for high-risk disease but is not currently endorsed for routine intraoperative decision-making. AI-assisted tumor modeling, PSMA-targeted fluorescence imaging, and digital confocal microscopy are not yet guideline-mandated and should be considered adjunctive or investigational tools rather than standards of care. Below is a table that demonstrates the different imaging and histologic modalities and their respective signal types, detection limits, time/cost factors and known pitfalls.

An important limitation of this review—and of the field more broadly—is the asymmetry in evidence maturity across technology-assisted strategies for optimizing robotic-assisted radical prostatectomy. NeuroSAFE remains the only intraoperative approach supported by randomized data, whereas AI-driven tools and PSMA-guided surgical strategies remain at earlier stages of clinical validation. The predominance of retrospective, single-institution studies raise concerns regarding selection bias, center-specific expertise, and overfitting, underscoring the need for prospective, multicenter trials with standardized endpoints and meaningful clinical outcomes.

**Figure 8:**
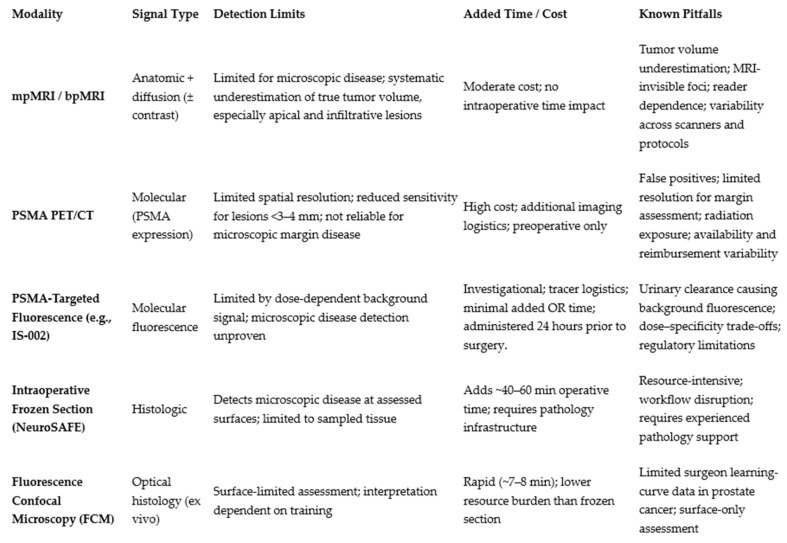
The table above contrasts the different imaging and histologic modalities, signal types, detection limits, time/cost factors and known pitfalls of various modalities.

Collectively, these modalities, summarized in Figure 8, illustrate an emerging multimodal surgical paradigm in which anatomic imaging, molecular targeting, and rapid histologic confirmation operate synergistically to guide resection planes, preserve critical structures, and optimize oncologic and functional outcomes. Until higher-level evidence is available, their integration into routine practice should be individualized, resource-aware, and guided by careful interpretation of evolving data.

## Data Availability

Not applicable. No new data were created or analyzed in this study.
